# Muscle Synergies Facilitate Computational Prediction of Subject-Specific Walking Motions

**DOI:** 10.3389/fbioe.2016.00077

**Published:** 2016-10-13

**Authors:** Andrew J. Meyer, Ilan Eskinazi, Jennifer N. Jackson, Anil V. Rao, Carolynn Patten, Benjamin J. Fregly

**Affiliations:** ^1^Department of Mechanical and Aerospace Engineering, University of Florida, Gainesville, FL, USA; ^2^Department of Biomedical Engineering, University of Florida, Gainesville, FL, USA; ^3^Department of Physical Therapy, University of Florida, Gainesville, FL, USA; ^4^Neural Control of Movement Lab, Malcom-Randall VA Medical Center, Gainesville, FL, USA

**Keywords:** biomechanics, computational neurorehabilitation, direct collocation optimal control, muscle synergy analysis, neuromusculoskeletal modeling, predictive gait optimization

## Abstract

Researchers have explored a variety of neurorehabilitation approaches to restore normal walking function following a stroke. However, there is currently no objective means for prescribing and implementing treatments that are likely to maximize recovery of walking function for any particular patient. As a first step toward optimizing neurorehabilitation effectiveness, this study develops and evaluates a patient-specific synergy-controlled neuromusculoskeletal simulation framework that can predict walking motions for an individual post-stroke. The main question we addressed was whether driving a subject-specific neuromusculoskeletal model with muscle synergy controls (5 per leg) facilitates generation of accurate walking predictions compared to a model driven by muscle activation controls (35 per leg) or joint torque controls (5 per leg). To explore this question, we developed a subject-specific neuromusculoskeletal model of a single high-functioning hemiparetic subject using instrumented treadmill walking data collected at the subject’s self-selected speed of 0.5 m/s. The model included subject-specific representations of lower-body kinematic structure, foot–ground contact behavior, electromyography-driven muscle force generation, and neural control limitations and remaining capabilities. Using direct collocation optimal control and the subject-specific model, we evaluated the ability of the three control approaches to predict the subject’s walking kinematics and kinetics at two speeds (0.5 and 0.8 m/s) for which experimental data were available from the subject. We also evaluated whether synergy controls could predict a physically realistic gait period at one speed (1.1 m/s) for which no experimental data were available. All three control approaches predicted the subject’s walking kinematics and kinetics (including ground reaction forces) well for the model calibration speed of 0.5 m/s. However, only activation and synergy controls could predict the subject’s walking kinematics and kinetics well for the faster non-calibration speed of 0.8 m/s, with synergy controls predicting the new gait period the most accurately. When used to predict how the subject would walk at 1.1 m/s, synergy controls predicted a gait period close to that estimated from the linear relationship between gait speed and stride length. These findings suggest that our neuromusculoskeletal simulation framework may be able to bridge the gap between patient-specific muscle synergy information and resulting functional capabilities and limitations.

## Introduction

Roughly one in six people worldwide will suffer a stroke at some point in their lifetime, with ~15 million people experiencing a stroke each year (World Stroke Organization, 2016). Due to improvements in acute stroke management, the majority of these individuals will survive their initial stroke, which has helped make stroke a leading cause of serious, long-term disability in adults worldwide (Go et al., 2013; World Stroke Organization, 2016). More than a third of stroke survivors experience significant physical disability (Lloyd-Jones et al., 2010), with walking dysfunction being among the greatest stroke-related limitations contributing to disability. While the majority of persons who suffer a stroke regain some level of ambulatory function, their gait is typically slow, asymmetrical, and metabolically inefficient (Olney et al., 1986; Roth et al., 1997). Diminished walking ability is tied to decreased quality of life, increased risk of depression, and increased risk of serious secondary health conditions (Blair et al., 1989; Mutikainen et al., 2011; Ostir et al., 2013). Restoration of walking function following a stroke is therefore both a high priority for rehabilitation and an important public health problem.

Despite recognition of the problem, current clinic-based neurorehabilitation methods produce only modest improvements in walking function for persons post-stroke (States et al., 2009; Bogey and Hornby, 2014; Winstein et al., 2016). For this reason, researchers and clinicians have explored a variety of neurorehabilitation approaches in search of an effective means to restore post-stroke walking function. These approaches include functional electrical stimulation (FES) (Popovic et al., 1999; Kesar et al., 2009, 2010; Sabut et al., 2013; Chung et al., 2014; O’Dell et al., 2014; Pilkar et al., 2014; Auchstaetter et al., 2015; Chantraine et al., 2016), ankle–foot orthoses (AFOs) (Ferreira et al., 2013; Tyson et al., 2013; Kobayashi et al., 2016), exoskeletons (Nilsson et al., 2014; Bortole et al., 2015; Buesing et al., 2015), partial body weight support (Ng et al., 2008; Lee et al., 2013) and split-belt treadmill training systems (Reisman et al., 2007; Malone and Bastian, 2014), and robotic gait trainers (Pennycott et al., 2012; Mehrholz et al., 2013; Bae et al., 2014; Hussain, 2014; Dundar et al., 2015). Each of these approaches has shown varying levels of promise for improving post-stroke walking function. However, a critical challenge is determining the treatment prescription – *which* approach to apply, *how much* of the approach to apply, and *how* the approach should be applied – that will maximize recovery of walking function for any particular individual. Furthermore, there is currently no way to identify whether a small amount of treatment provided by a combination of approaches might be dramatically more effective than a large amount of treatment provided by a single approach (Belda-Lois et al., 2011). Current treatment design methods based on trial-and-error and subjective clinical judgment cannot address these challenges, since they do not provide an objective means for predicting a patient’s walking function following a specified treatment or treatment combination.

One possible approach for overcoming this challenge is to use patient-specific neuromusculoskeletal models to predict post-treatment walking function for different neurorehabilitation technologies (alone or combined) under consideration. Such models should account for how the patient interacts with the treatment approach (Mooney and Herr, 2016) so that the optimal prescription can be recommended based on objective predictions of walking improvement. A number of studies have pursued such modeling efforts by simulating the effects of FES (Riener, 1999; Heilman and Kirsch, 2003; Zhang and Zhu, 2007; Shao and Buchanan, 2008; Nekoukar and Erfanian, 2013; Sharma et al., 2014; Alibeji et al., 2015), exoskeletons (Fleischer and Hommel, 2008; Afschrift et al., 2014; Farris et al., 2014; Sawicki and Khan, 2015), orthoses (Zmitrewicz et al., 2007; Crabtree and Higginson, 2009; Silverman and Neptune, 2012), and strength training (Goldberg and Neptune, 2007; Knarr et al., 2014) on lower extremity function and walking ability in the context of stroke, spinal cord injury, amputee, and general rehabilitation. Few of these studies focused on stroke (Goldberg and Neptune, 2007; Shao and Buchanan, 2008; Knarr et al., 2014), few used three-dimensional models (Fleischer and Hommel, 2008; Afschrift et al., 2014; Farris et al., 2014; Knarr et al., 2014; Sawicki and Khan, 2015), few used subject-specific models created by calibrating critical neuromusculoskeletal model parameters to movement data collected from an individual (Fleischer and Hommel, 2008; Shao and Buchanan, 2008; Knarr et al., 2014), and only one included modeling elements that accounted for subject-specific neural control capabilities and limitations (Alibeji et al., 2015). No study to date has predicted a stroke patient’s complete post-treatment walking motion and speed resulting from application of a specific neurorehabilitation intervention.

As a first step toward optimizing patient interaction with stroke neurorehabilitation technologies, this study describes the development and evaluation of a subject-specific synergy-controlled neuromusculoskeletal simulation framework that can predict three-dimensional walking motions for an individual post-stroke. The main question we address is whether actuating a subject-specific neuromusculoskeletal model with muscle synergy controls (5 per leg) facilitates generation of accurate walking predictions compared to actuating the model with muscle activation controls (35 per leg) or joint torque controls (5 per leg). We hypothesize that synergy controls will work the best since they combine a low number of control signals with a subject-specific representation of the coupling between muscle activations within each leg. We collect gait data from a stroke subject walking at 0.4, 0.5, 0.6, 0.7, and 0.8 m/s on an instrumented treadmill and use data from his self-selected speeds of 0.4–0.6 m/s to develop a subject-specific neuromusculoskeletal model. We incorporate the subject-specific full-body model into a direct collocation optimal control framework to predict new walking motions for the subject. To evaluate the framework and the potential benefits of using synergy controls, we predict how the individual will walk (including cadence and stride length) at 0.5 and 0.8 m/s (conditions for which experimental data are available for comparison) using joint torque, muscle activation, or muscle synergy controls and at 1.1 m/s (a condition for which no experimental data are available) using only synergy controls. With future simulation of different neurorehabilitation approaches, our subject-specific synergy-controlled neuromusculoskeletal simulation framework may help identify optimal neurorehabilitation prescriptions that maximize recovery of walking function on an individual patient basis.

## Methods

### Experimental Data Collection

To assist with development and evaluation of our subject-specific synergy-controlled neuromusculoskeletal simulation framework, we collected experimental walking data from one high-functioning hemiparetic male individual with chronic stroke-related walking dysfunction (age 79 years, LE Fugl-Meyer Motor Assessment 32/34 pts, right-sided hemiparesis, height 1.7 m, mass 80.5 kg). All study procedures were approved by the University of Florida Health Science Center Institutional Review Board (IRB-01) and the Malcom Randall VA Medical Center Research and Development Committee and included approval to study individuals with stroke-related disability. Study personnel obtained written informed consent prior to participant enrollment and involvement in study activities. Study procedures were conducted in accordance with the Declaration of Helsinki. Motion capture (Vicon Corp., Oxford, UK), ground reaction (Bertec Corp., Columbus, OH, USA), and electromyography (EMG) data (Motion Lab Systems, Baton Rouge, LA, USA) were collected simultaneously while the participant walked on a split-belt instrumented treadmill (Bertec Corp., Columbus, OH, USA) at speeds ranging from 0.4 to 0.8 m/s in increments of 0.1 m/s. 0.8 m/s was the fastest speed at which the subject could walk safely without assistance. This range of speeds included the participant’s self-selected walking speed of 0.5 m/s. More than 50 gait cycles were recorded at each walking speed. A static standing trial was collected for model scaling purposes. To facilitate subsequent creation of subject-specific foot–ground contact models, the participant wore Adidas Samba Classic sneakers, which have a flat sole and neutral midsole with no cushioning, and we collected additional static trials where we used a marker wand to trace the outline of each sneaker sole on the ground. Motion capture data were obtained using a modified Cleveland Clinic full-body marker set with additional markers added to the feet (Reinbolt et al., 2005). Marker motion and ground reaction data were filtered at a variable cut-off frequency of 7/tf Hz, where tf is the period of the gait cycle being processed, using a fourth-order zero phase lag Butterworth filter (Hug, 2011). This variable cut-off frequency would cause data collected at a normal walking speed to be filtered at ~6 Hz.

Electromyography data were collected from 16 muscles in each leg and processed using standard methods (Lloyd and Besier, 2003). A combination of surface and fine-wire EMG electrodes was used. Surface EMG data were collected for gluteus maximus and medius, semimembranosus, biceps femoris long head, rectus femoris, vastus medialis and lateralis, medial gastrocnemius, tibialis anterior, peroneus longus, and soleus. Fine-wire EMG data were collected for adductor longus, iliopsoas, tibialis posterior, extensor digitorum longus, and flexor digitorum longus. All EMG data were high-pass filtered at 40 Hz (Lloyd and Besier, 2003), demeaned, rectified, and then low-pass filtered at a variable cut-off frequency 3.5/tf Hz. Filtering was performed using a fourth-order zero phase lag Butterworth filter. EMG data from each muscle were normalized to the maximum value over all trials and resampled to 101 time points per gait cycle (heel strike to heel strike for the less involved left side) while keeping an additional 20 time points before the start of the cycle to permit modeling of electromechanical delay. In addition, each processed EMG signal was offset on a cycle-by-cycle basis so that the minimum value was zero.

### Neuromusculoskeletal Model Development

The subject-specific neuromusculoskeletal model that served as the foundation for our simulation framework incorporated four modeling components to account for the unique neurophysiological and musculoskeletal characteristics of the subject: (1) a subject-specific lower-body kinematic model to simulate the subject’s skeletal motion, (2) subject-specific foot–ground contact models to simulate how the subject’s feet interact with the ground, (3) subject-specific EMG-driven muscle moment models to simulate the subject’s lower extremity joint moments, and (4) a subject-specific muscle synergy control model to simulate the subject’s neural control system. Below we describe each of these four components in further detail. Unless otherwise noted, we calibrated model parameters in each component using a single representative walking trial collected at the subject’s self-selected speed of 0.5 m/s.

#### Subject-Specific Lower-Body Kinematic Model

Our neuromusculoskeletal model creation process started with a generic full-body musculoskeletal model (Arnold et al., 2010; Hamner et al., 2010) developed in OpenSim (Delp et al., 2007). The original model included 37 degrees of freedom (DOFs) and 44 Hill-type muscle-tendon actuators in each leg. We locked the wrist and forearm pronation–supination angles to anatomically reasonable values for walking, leaving the following 31 DOFs: 6 DOF ground-to-pelvis joint, 3 DOF hip joints, 1 DOF knee joints, 1 DOF ankle joints, 1 DOF subtalar joints, 1 DOF toe joints connecting rear foot and toe segments, 3 DOF back joint, 3 DOF shoulder joints, and 1 DOF elbow joints. We also eliminated nine muscle-tendon actuators without related EMG data (extensor hallucis longus, flexor hallucis longus, gemelli, gracilis, pectineus, piriformis, quadratus femoris, sartorius, tensor fascia latae), leaving 35 muscles per leg that actuated hip flexion-extension, hip adduction-abduction, knee flexion-extension, ankle flexion-extension, and ankle inversion–eversion on each leg. We then scaled the modified model using the standing static trial marker data and the OpenSim “Scale Model” tool, where distances between markers placed over identifiable landmarks were averaged between the two sides to maintain bilateral symmetry following scaling.

Once the model was scaled, we calibrated joint and marker positions in the torso, pelvis, and lower-body portion of the OpenSim model using marker data from a representative walking trial. The calibration approach was similar to one described previously (Reinbolt et al., 2005, 2008) except that it was performed on the scaled OpenSim model using the OpenSim-MATLAB Application Programing Interface and included modifications to maintain correct bone geometry positions within the body segments (Charlton et al., 2004). To facilitate the calibration process, we created marker plates on the torso, pelvis, thighs, and shanks to which all markers on the respective OpenSim body segments were attached. To perform the actual calibration, we used non-linear least squares optimization (lsqnonlin) in MATLAB to adjust joint (knee, ankle, and subtalar in both legs) and marker plate (torso, pelvis, thighs, and shanks) positions and orientations in their respective body segments such that marker errors from repeated OpenSim inverse kinematic analyses were minimized. The optimization cost function included penalty terms that prevented large changes in joint and marker plate positions and orientations that would produce only small improvements in marker tracking. Modification of the two hip joint center locations was achieved by modifying the position and orientation of the rigid marker plate on the pelvis. For joint centers and orientations, symmetry between left and right sides of the body was maintained during the kinematic calibration process. Markers on the feet were not adjusted since their locations were well defined. The position and orientation of the toe axis in each foot and of the back, shoulder, and elbow joints was maintained from the scaled OpenSim model.

#### Subject-Specific Foot–Ground Contact Models

Following kinematic calibration, we created a subject-specific foot–ground contact model for each foot of the OpenSim model using recently developed methods (Jackson et al., 2016). The elastic foundation contact models were developed in MATLAB and used a grid of contact elements that spanned the rear foot and toes segments of each foot. To create the element grid, we started with the shoe outlines obtained from the static trial marker data and used principal component analysis to identify the principal axes of each foot (rear foot and toes segments together). Using these axes, we constructed an 11 (anterior-posterior) × 8 (medial-lateral) grid of rectangular contact elements for the left foot, where the edges of the grid extended 2.5 mm beyond the edge of the shoe outline in both directions. Forty-seven elements whose centers fell within the shoe outline were retained in the contact model, while 41 elements whose centers fell outside the shoe outline were removed. Given the locations of the MATLAB contact element centers relative to the foot markers from the static left shoe outline trial, we calculated the locations of the element centers on the OpenSim rear foot and toes segments. We then projected the left toes axis of the OpenSim model onto the contact element grid. Elements whose centers were posterior to the axis were assigned to the rear foot segment, while elements whose centers were anterior to the axis were assigned to the toes segment. The complete MATLAB/OpenSim contact element grid for the left foot was mirrored to the right foot by aligning the principal axes of the mirrored grid with those of the right foot.

Each contact element in the foot–ground contact models generated normal force using a linear spring with non-linear damping and shear force using a continuous stick-slip friction model. For any contact element *i*, the required time-varying inputs for contact force calculations performed in MATLAB were the penetration into the floor *y_i_*, the normal penetration rate ${\dot{y}}_{i}$, and the shear slip rate $v_{s_{i}}$ of the element center in the laboratory coordinate system as calculated by OpenSim. The normal contact force *F_i_* for element *i* was calculated as (Hunt and Crossley, 1975)

(1) $$F_{i}=k_{i}(y_{i}-y_{0})(1+c{\dot{y}}_{i})$$

where *k_i_* is the spring stiffness unique to each spring, *y*_0_ is the spring resting length common to all springs on the same foot and essentially adjusts the height of the floor, and *c* (= 1e–2) is a non-linear damping coefficient common to all springs. The linear spring also generates a small amount of force when the foot is off the floor, and this negligible force transitions in a smooth and continuous way to the large force produced when the spring is in contact with the ground (Anderson and Pandy, 2001; Ackermann and van den Bogert, 2010). The non-linear damping ensures that the normal contact force does not exhibit damping-related discontinuities when a spring enters or leaves contact. The shear contact force *f_i_* for element *i* was calculated using a simple continuous and differentiable friction model (Ackermann and van den Bogert, 2010)

(2) $$f_{i}=F_{i}\left[\mu \tanh\left(\frac{v_{s_{i}}}{v_{l}}\right)\right]$$

where μ [= 1 (Ackermann and van den Bogert, 2010)] is a dynamic friction coefficient common to all springs and *v_l_* (= 5 cm/s) is a latching speed common to all springs that defines the edge of a linear transition region between zero slip rate and the start of dynamic friction. Shear contact force *f_i_* was applied to the element center in the direction opposite to the slip velocity vector. The direction calculation included a small constant value of 1e−4 in the denominator to avoid division by a small number when the slip speed was near zero. Once *F_i_* and *f_i_* were calculated for each contact element, the net contact force and torque due to all contact elements in the rear foot segment were calculated with respect to the rear foot origin, and similarly for the toes segment using the toes origin (Kane and Levinson, 1985). These net contact forces and torques were then applied to their corresponding segments in the OpenSim model. This approach allowed rear foot and toes contributions to total ground reaction force to be predicted by the model.

We calibrated the spring stiffness *k_i_* of each contact element in both feet and the spring resting length *y*_0_ for all contact elements in each foot using marker and ground reaction data from a representative walking trial. We made two assumptions about the spring stiffness distribution across the bottom of the shoe to simplify the calibration process. First, we assumed that the mirrored stiffness distribution was the same for both feet. Second, we assumed that the stiffness distribution across the entire shoe bottom could be approximated by a three-dimensional parabolic surface, which possesses only six unknown coefficients rather than 47 unknown independent spring stiffness values and prevents neighboring springs from having dramatically different stiffnesses. To calibrate these six coefficients and the two resting lengths, we formulated a direct collocation optimal control problem that tracked experimental marker, ground reaction, and inverse dynamic joint torque data for the entire body with higher weight placed on matching marker position and ground reaction data for the two feet. Tracked ground reaction quantities for each foot included the three ground reaction force components and three ground reaction torque components calculated about the midfoot marker projected onto the floor (see Optimal Control Walking Predictions below for further details).

#### Subject-Specific EMG-Driven Muscle Moment Models

To generate subject-specific joint moments from predicted muscle activations, we calibrated lower extremity EMG-driven muscle moment models for both legs to a large number of walking trials collected from the subject. Complete details of our EMG-driven model calibration process, and a full assessment of its ability to predict joint moments accurately for the same subject walking at multiple speeds, can be found in Meyer et al. (2016). In brief, the model calibration process used experimental walking data collected from the subject at 0.4, 0.5, and 0.6 m/s, bracketing his self-selected speed of 0.5 m/s. Ten trials from each speed were used for calibration. We adjusted three types of model parameter values in our calibration process: (1) EMG-to-activation parameter values, (2) Hill-type muscle-tendon model parameter values, and (3) surrogate musculoskeletal geometry parameter values. Below we describe each category of adjusted model parameter values in greater detail.

For our EMG-to-activation model, parameter values adjusted during calibration included electromechanical delays, EMG scale factors, activation time constants, and muscle non-linearity constants. A single electromechanical time delay between 0 and 100 ms was used for all muscles in the same leg, allowing the two legs to have different electromechanical delays. An EMG scale factor was found for each muscle in each leg, resulting in 70 different scale factors. An activation time constant for a first-order activation dynamics model (He et al., 1991) was found for each muscle, with time constants assumed to be identical for the same muscle in both legs, resulting in 35 different time constants. Deactivation time constants were assumed to be four times larger than corresponding activation time constants (Thelen, 2003; De Groote et al., 2012; Millard et al., 2013). Finally, a non-linear constant defining the conversion of neural activation to muscle activation was found for each muscle (Buchanan et al., 2004), with non-linear constants assumed to be identical for the same muscle in the two legs, resulting in 35 different non-linear constants.

For our Hill-type muscle-tendon model, parameter values adjusted during calibration included optimal muscle fiber lengths and tendon slack lengths. We used a custom Hill-type muscle-tendon model with a rigid tendon, as a recent study has shown that use of a compliant tendon model for simulations of walking has little effect on predicted muscle activations and forces (De Groote et al., 2016). This model was implemented in MATLAB to facilitate customization of model properties. Initial Hill-type model parameter values (optimal muscle fiber length, tendon slack length, pennation angle) for each muscle were taken from the literature (Arnold et al., 2010) and assumed to be the same for both legs. Optimal fiber length and tendon slack length values were pre-calibrated to reproduce passive hip, knee, and ankle flexion moment data reported in the literature (Silder et al., 2007). Peak isometric force for each muscle was defined using regression equations for muscle volume reported in the literature (Handsfield et al., 2014) along with each muscle’s optimal fiber length and a maximum muscle stress of 61 N/cm^2^ (Arnold et al., 2010). Maximum shortening velocity for each muscle was defined to be 10 optimal fiber lengths per second.

For our surrogate musculoskeletal geometry, parameter values adjusted during calibration included coefficients of polynomial functions defining muscle-tendon lengths as a function of spanned joint angles. For each muscle-tendon actuator in our kinematically-calibrated OpenSim model, we first created a surrogate model of muscle-tendon length using a cubic polynomial function of all spanned joint angles. Some muscles required a cubic function of only one joint angle (e.g., vastus medialis), while other muscles required a cubic function of multiple joint angles (e.g., gluteus maximus and gastrocnemius medialis). We then created corresponding surrogate models of muscle-tendon velocity and moment arms by defining muscle-tendon velocity as the first derivative of the muscle-tendon length polynomial with respect to time and each muscle moment arm as the negative of the first derivative of the muscle-tendon length polynomial with respect to the corresponding spanned joint angle (An et al., 1984). In this way, the polynomial functions defining muscle-tendon lengths, velocities, and moment arms shared common coefficients (Menegaldo et al., 2004; Sartori et al., 2012a).

To generate an initial polynomial fit for each muscle, we sampled muscle-tendon lengths and moment arms from our kinematically calibrated OpenSim model using a wide range of lower extremity joint angle combinations. The maximum and minimum value of each joint angle were allowed to go well beyond the values achieved by the subject during walking. Sampling was performed using 1000 different model poses specified using a Latin hypercube design, with muscle-tendon lengths and moment arms being calculated by an OpenSim “Muscle Analysis.” After outliers were removed corresponding to situations with muscle wrapping problems, we used linear least squares regression to fit muscle-tendon length and moment arms for each muscle as a cubic polynomial function of spanned joint angles.

We used sequential quadratic programing (SQP) optimization (fmincon) in MATLAB to adjust the parameter values described above such that EMG-driven models for both legs matched lower extremity inverse dynamic and passive joint moment curves as closely as possible. Inputs to the optimization were the subject’s processed experimental EMG data, joint kinematics from OpenSim “Inverse Kinematics” analyses, and joint moments from OpenSim “Inverse Dynamics” analyses from 30 selected walking trials, along with the published passive joint moment data described earlier (Silder et al., 2007). For modeled muscles without experimental EMG data (e.g., vastus intermedius) or with multiple compartments (e.g., gluteus maximus), EMG data from anatomically related muscles were used but with a separate scale factor for each muscle/compartment (Sartori et al., 2012b). Outputs were the model parameter values and predicted inverse dynamic and passive joint moments. The subject’s hip internal–external rotation moment from walking was not included in the calibration process, since EMG data were not collected from primary hip external rotator muscles. Thus, the EMG-driven model for each leg matched five inverse dynamic moments (hip flexion–extension, hip adduction–abduction, knee flexion–extension, ankle flexion–extension, and ankle inversion–eversion). During calibration, optimal muscle fiber length and tendon slack length values were allowed to vary within 25% of the values produced by pre-calibration, while changes in surrogate musculoskeletal geometry were strongly penalized so that such changes would be made only if they resulted in significant improvements in joint moment matching. The calibrated EMG-driven model for each leg was verified by predicting lower extremity joint moments for walking trials withheld from calibration, including trials from faster walking speeds. These models were used in all subsequent activation- and synergy-driven optimal control simulations.

#### Subject-Specific Muscle Synergy Control Model

The standard method of performing muscle synergy analysis uses only processed experimental EMG data. Following processing, EMG data for one complete walking cycle are organized into a matrix, and non-negative matrix factorization is applied via an optimization approach to decompose the high dimensional set of processed EMG signals into a lower dimensional set of time-varying signals (which we will call “synergy controls”) with associated sets of muscle weights (which we will call “synergy vectors”) (Lee and Seung, 1999; Tresch et al., 1999). The synergy vector weights specify how each synergy control contributes to each processed EMG signal. Each time a synergy analysis is performed, the number of synergies to fit must be specified *a priori*. Typically only three to six synergy controls are needed to reconstruct a much larger number of processed EMG signals with a high “variability account for” (VAF), typically above 90% (Ivanenko et al., 2005; Clark et al., 2010).

There are at least two drawbacks to the standard synergy analysis approach. First, the absolute amplitude of each processed EMG signal remains unknown. Though EMG signals are commonly normalized using data from a maximum voluntary contraction trial or the movement trial with maximum signal amplitude, maximal M-wave measurements reveal that these methods do not yield the true maximum EMG value (Clark et al., 2006; Simonsen et al., 2012; Racinais et al., 2013; Cronin et al., 2015). Since EMG amplitudes affect the results of a muscle synergy analysis, this issue makes it difficult to use experimentally derived synergy information for musculoskeletal modeling purposes. Second, since standard synergy analysis only uses experimental EMG data, it does not provide any information on how the inter-muscle coupling quantified by the synergy vectors affects an individual’s ability to perform specific movement tasks. Thus, a gap exists between subject-specific neural control information provided by standard muscle synergy analysis and the functional consequences of that information.

To bridge this gap while also addressing the EMG normalization issue, we perform muscle synergy analysis for our subject within the larger context of producing a dynamically consistent full-body walking motion using a subject-specific neuromusculoskeletal model (Sharif Razavian et al., 2015). With standard synergy analysis, the goal is to find a specified number of synergy controls and vectors that best match a larger set of normalized muscle EMG signals. With our approach, the goal is to find a specified number of synergy controls and vectors that best match experimental joint motions, ground reactions, lower-body inverse dynamic joint moments, and scaled EMG signals produced by our EMG-driven models. Thus, the breadth of data matched by our approach is much larger than that of a standard muscle synergy analysis. We describe our expanded approach as “dynamically consistent synergy analysis.” Use of EMG-driven models within our neuromusculoskeletal model provides a unique way to address the EMG normalization issue, while finding dynamically consistent full-body walking motions with our neuromusculoskeletal model bridges the gap between neural control information and its functional consequences.

To find synergy controls and vectors that could reproduce our subject’s walking data at his self-selected speed of 0.5 m/s, we followed a two-step process. First, we performed standard synergy analysis on the 35 muscle activations for each leg produced by the EMG-driven model calibration process for a representative walking trial. We performed this step on muscle activations rather than scaled EMG signals since we omitted activation dynamics from our final neuromusculoskeletal model to reduce model complexity. We incremented the number of synergies found by standard synergy analysis until the total VAF was greater than 95% and the VAF for each muscle was greater than 85%. We chose these high values since our goal was not simply reconstruction of activations but also reproduction of a dynamically consistent walking motion. Five synergies were required to achieve the target VAF values. Second, we performed a tracking optimization using direct collocation optimal control where all muscles in each leg were driven by five synergy controls. The synergy controls and associated synergy vectors were unknowns to be found by the optimization. The optimization tracked ground reactions, muscle activations, lower-body joint torques, and upper body joint motions while producing a dynamically consistent walking motion (see Optimal Control Walking Predictions below for further details).

### Optimal Control Walking Predictions

We used the subject-specific neuromusculoskeletal model described above and direct collocation optimal control to predict the subject’s walking motion at 0.5 and 0.8 m/s given walking data from the most periodic trial collected at his self-selected speed of 0.5 m/s. A key advantage of collocation methods over shooting methods is that they use implicit rather than explicit simulation. Repeatedly during the non-linear programing (NLP) solution process, shooting methods perform explicit simulation to solve the system dynamics sequentially for one time frame at a time via numerical integration. This process is often unstable because either errors accumulate with each integration step or the system being simulated is inherently unstable, as with the human body during walking. By contrast, as part of the NLP solution process, collocation methods perform implicit simulation to solve the system dynamics for all time frames simultaneously with no notion of time stepping. Consequently, instabilities arising from accumulated integration errors or inherent system instabilities are eliminated, facilitating the use of gradient-based optimization for predicting motion. Another advantage of collocation is that feedback control, which is artificially introduced in explicit simulation to maintain system stability, is unnecessary since time stepping is not performed by implicit simulation. An overview of various numerical methods for solving optimal control problems, along with a brief discussion of the advantages and disadvantages of each, can be found in Limebeer and Rao (2015).

We investigated how well the predictions worked using three different control situations: joint torque controls (5 per leg – termed “torque-driven”), muscle activation controls (35 per leg – termed “activation-driven”), and muscle synergy controls (5 per leg – termed “synergy-driven”). To control the motion of each leg, the torque-driven problems used 5 joint torques rather than 35 muscles, the activation-driven problems used 35 muscles controlled by 35 independent muscle activations, and the synergy-driven problems used 35 muscles controlled by 5 independent muscle synergy controls that were linearly combined to produce 35 muscle activations. For all three control situations, hip internal–external rotation and toes flexion–extension in both legs along with the three pelvis rotations were found by tracking the corresponding joint angles from the 0.5 m/s periodic trial.

To generate our predictions, we performed a sequence of three categories of optimizations: (1) calibration optimizations, (2) tracking optimizations, and (3) prediction optimizations (see Table 1 for overview). This sequence was needed since large-scale direct collocation optimal control problems are often sensitive to the initial guess, making it helpful to increase the complexity of the problems being solved in a gradual and systematic fashion. Furthermore, for each category of optimization, initial problems were solved where the skeletal dynamic constraints (as quantified by pelvis residual loads) were not enforced, and these constraints were gradually tightened in subsequent problems until the desired tolerance was met. Below we describe concepts common to all three optimization categories, integration of OpenSim functionality into the optimal control framework, and details for the three optimization categories.

**Table 1:** **Sequence of direct collocation optimal control problems solved using GPOPS-II to predict patient-specific walking motions at 0.5, 0.8, and 1.1 m/s**.

	Cost Function	Constraints	Static Parameters	Controls
**1 Calibration Optimizations**				
1.1 Torque-driven model	Track experimental marker, ground reaction, and inverse dynamic torque data; Minimize joint jerk	Satisfy skeletal dynamics	Foot–ground contact model parameters	Joint jerk
1.2 Torque-driven model	Track experimental marker, ground reaction, and inverse dynamic torque data; Minimize joint jerk	Satisfy skeletal dynamics	None	Joint jerk
**2 Tracking Optimizations**				
2.1 Torque-driven model	Track lower-body joint torques and upper body joint angles from problem 2; Minimize joint jerk	Satisfy skeletal dynamics; Match OpenSim lower-body joint torques using torque controls; Bound joint angle errors relative to problem 2 and ground reaction errors relative to experimental data	None	Joint jerk; Joint torques
2.2 Activation-driven model	Track lower-body activation data; Track lower-body joint torques and upper body joint angles from problem 2; Minimize joint jerk	Satisfy skeletal dynamics; Match OpenSim lower-body joint torques using activation controls; Bound joint angle errors relative to problem 2 and ground reaction errors relative to experimental data	None	Joint jerk; Muscle activations
2.3 Synergy-driven model	Track lower-body activation data; Track lower-body joint torques and upper body joint angles from problem 2; Minimize joint jerk	Satisfy skeletal dynamics; Match OpenSim lower-body joint torques using synergy controls; Bound joint angle errors relative to problem 2 and ground reaction errors relative to experimental data; Enforce unit magnitude synergy vectors	Synergy vector weights	Joint jerk; Synergy controls
**3 Prediction optimizations**				
3.1 Torque-driven model	Track lower-body joint torques and upper body joint angles from problem 3; Minimize joint jerk	Satisfy skeletal dynamics; Match OpenSim lower-body joint torques using torque controls	None	Joint jerk; Joint torques
3.2 Activation-driven model	Track lower-body activations and upper body joint angles from problem 4; Minimize joint jerk	Satisfy skeletal dynamics; Match OpenSim lower-body joint torques using activation controls	None	Joint jerk; Muscle activations
3.3 Synergy-driven model	Track lower-body synergy controls and upper body joint angles from problem 5; Minimize joint jerk	Satisfy skeletal dynamics; Match OpenSim lower-body joint torques using synergy controls	None	Joint jerk; Synergy controls

*All optimizations were performed using a full-body patient-specific neuromusculoskeletal model with calibrated joint, musculoskeletal geometry, and muscle-tendon model parameter values (see text). Tracking optimizations were performed at 0.5 m/s to develop dynamically consistent baseline data for each of the three lower extremity control situations (joint torques, muscle activations, or muscle synergies) used in subsequent prediction optimizations. Prediction optimizations were performed for 0.5 m/s to verify optimal control problem formulation, 0.8 m/s to evaluate problem formulation, and 1.1 m/s to challenge problem formulation. Activations employ 35 controls per leg while synergies employ 5 controls per leg. Note that Problem 1.1 serves as the basis for Problem 1.2, Problem 1.2 serves as the basis for Problems 2.1, 2.2, and 2.3, and Problems 2.1, 2.2, and 2.3 serve as the basis for Problems 3.1, 3.2, and 3.3, respectively*.

#### Common Concepts

The walking predictions reported in this study were generated using GPOPS-II, a direct collocation optimal control toolbox for MATLAB (Patterson and Rao, 2014). GPOPS-II solves for the state *x*(*t*), control *u*(*t*), and static parameters *p* that minimize the cost functional:

(3) $$J=\phi \left(x(t_{0}),t_{0},x(t_{f}),t_{f},p\right)+\int_{t_{0}}^{t_{f}}g(x(t),u(t),p)dt$$

subject to the constraints

(4) $$\dot{x}(t)=f(x(t),u(t),t,p)\text{, }\left(\text{dynamic constraints}\right),$$

(5) $$c_{\min}\leq f\left(x(t),u(t),t,p\right)\leq c_{\max},\;\left(\text{algebraic constraints}\right),$$

(6) $$b_{\min}\leq b(x(t_{0}),t_{0},x(t_{f}),t_{f},p)\leq b_{\max},\;\left(\text{boundary conditions}\right).$$

The state and control are parameterized using variable-order Gaussian quadrature orthogonal collocation methods and formulated into a NLP problem.

Within the toolbox, two NLP solvers can be utilized: SNOPT (Gill et al., 2005) or IPOPT (Biegler and Zavala, 2009). SNOPT employs a quasi-Newton SQP active set method where the inverse of the Hessian is approximated using a Broyden–Fletcher–Goldfarb–Shanno (BFGS) update. At each step in the optimization, all linear constraints are satisfied and the active set is estimated. SNOPT achieves convergence via a merit function that is the objective function plus the sum of constraint infeasibilities. IPOPT employs an interior-point method where the goal is to satisfy the constraints via a barrier function. IPOPT does not distinguish between linear and non-linear constraints; all constraints are treated the same. IPOPT operates in two different modes. In first derivative mode, IPOPT employs a quasi-Newton method where the inverse of the Hessian is estimated using a BFGS update. In second derivative mode, IPOPT employs a full-Newton method where GPOPS-II provides a sparse representation of the lower triangular part of the Hessian of the Lagrangian of the NLP problem. Based on convergence and computation time considerations, we chose IPOPT in first derivative mode as the initial NLP solver for all optimal control problems. Though GPOPS-II contains an adaptive mesh refinement algorithm, we used a fixed mesh of 50 collocation points, divided into 10 intervals, over the entire gait cycle to reduce computation time (Ackermann and van den Bogert, 2010).

In this study, we developed a jerk-controlled inverse dynamic problem formulation using the 31 DOF skeletal dynamic equations generated by OpenSim (Delp et al., 2007). This approach was used since it exhibited improved convergence properties and solution smoothness over a directly controlled forward dynamic problem formulation (van den Bogert et al., 2011), but at the cost of added joint jerk controls. It also allowed us to violate the skeletal dynamics when starting from a poor initial guess, facilitating finding a feasible solution. For this problem formulation, we defined the control *u*(*t*) as the third time derivative of the generalized coordinates *q*(*t*) (i.e., joint jerk). The problem state consisted of the coordinate positions *q*(*t*), velocities *v*(*t*), and accelerations *a*(*t*), simplifying the dynamics of the optimal control problem to:

(7) $$\left[\begin{array}{c} \dot{q}\\ \dot{v}\\ \dot{a} \end{array}\right]=\left[\begin{array}{c} v\\ a\\ u \end{array}\right]$$

To make the joint jerk controls unique, we added minimization of joint jerk to every optimal control problem. Jerk minimization terms in the cost function were scaled by $t_{f}^{6}$, where *t_f_* is the specified final time, since jerk magnitude is proportional to $t_{f}^{3}$ based on analysis of analytic functions, and joint jerk squared is minimized in the cost function. Since joint jerk magnitudes change with final time, we did not want jerk minimization to affect the final times predicted by our optimizations.

For our inverse dynamic problem formulation, enforcement of the skeletal dynamics was achieved using algebraic path constraints. Two types of path constraints were used for this purpose. The first type ensured that residual forces and torques acting on the pelvis were eliminated. Each time an inverse dynamic analysis was performed with the OpenSim model, six pelvis residual loads *R*_pelvis_(*x*(*t*)) were calculated. To enforce dynamic consistency, we added the algebraic path constraint

(8) $$R_{\text{min}}\leq R_{\text{pelvis}}\leq R_{\text{max}}$$

to constrain the six pelvis residual loads to be within a specified tolerance. The second type of constraint enforced consistency between net muscle moments calculated from additional controls and lower extremity joint moments calculated from inverse dynamics. The additional controls were 5 joint torques per leg for torque-driven problems, 35 activations per leg for activation-driven problems, 5 synergies per leg for synergy-driven problems. For all three control situations, we added algebraic path constraints to ensure that the additional controls balanced five lower extremity inverse dynamic joint moments in each leg (i.e., hip flexion–extension, hip adduction–abduction, knee flexion–extension, ankle flexion–extension, and ankle inversion–eversion). Convergence tolerances were set to 1 N and 0.1 Nm for residual forces and moments, respectively. When this tolerance was met, we considered the resulting motion to be dynamically consistent, requiring negligible fictitious external loads to balance the dynamic equations. All optimal control solutions presented in this study utilized this inverse dynamic problem formulation. However, the cost function, controls, and constraints varied depending on the category of optimization problem being solved.

#### OpenSim Integration

**Figure 1:**
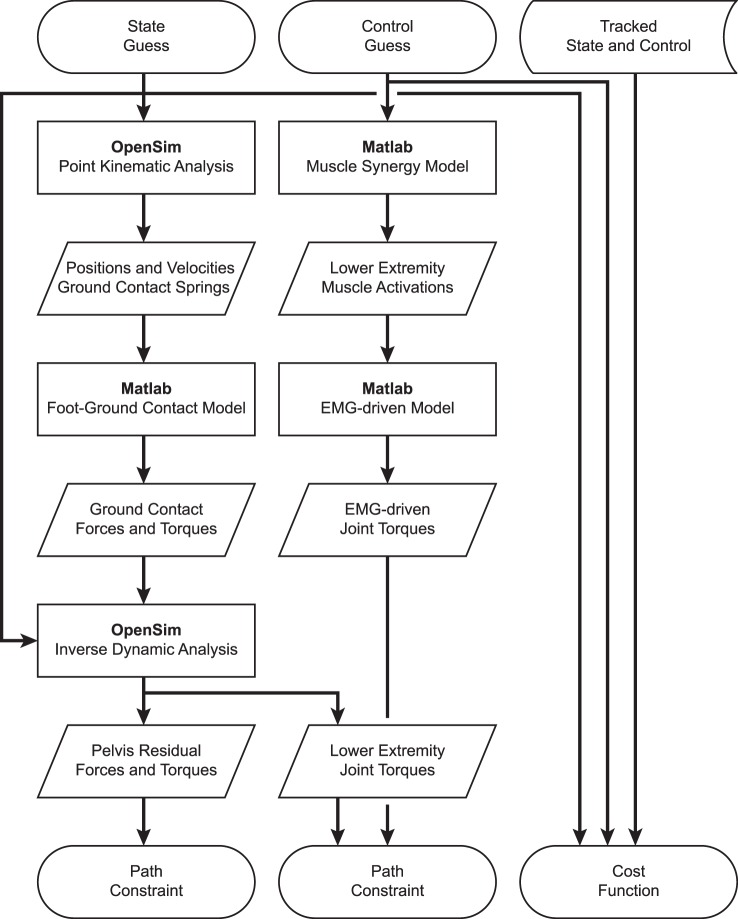
**Flowchart demonstrating the interaction of MATLAB and OpenSim functions for synergy-driven prediction optimizations performed within the GPOPS-II MATLAB environment**. For each iteration of the NLP solver, GPOPS-II provides a new guess for the model state and control, which are used by a series of MATLAB and OpenSim functions to calculate path constraints, terminal constraints (not shown – used to enforce near-periodicity in motion and ground reactions), and the cost function. The cost function requires knowledge of state and control quantities that are being tracked, which are taken from the results of the corresponding tracking optimization. Path constraints are used to satisfy skeletal dynamics and to make lower-body joint moments calculated by an OpenSim inverse dynamic analysis match corresponding joint moments calculated by a MATLAB EMG-driven model. Synergy-driven prediction optimizations use five synergy controls per leg as controls. Activation-driven prediction optimizations use 35 muscle activations per leg as controls and do not require the MATLAB muscle synergy model. Torque-driven prediction optimizations use five joint torques per leg as controls and do not require the MATLAB muscle synergy model or EMG-driven model. Calibration optimizations include additional path constraints to match additional experimental quantities (e.g., ground reaction forces and moments).

Development of dynamically consistent full-body walking predictions required integrating OpenSim functionality into the MATLAB environment in a computationally efficient manner (Figure 1). To perform the integration, we took advantage of two aspects of our optimal control solution process. First, use of joint jerk controls made complete OpenSim model state and state derivative information available for any time point being analyzed, making it possible to evaluate the OpenSim skeletal dynamic equations in an inverse sense and avoiding the need to calculate OpenSim model state derivatives for forward solutions. Second, use of a direct collocation solution approach allowed each time point to be analyzed independently from all others, making it possible to employ parallel processing methods on a time-point-by-time-point basis.

To take advantage of these two aspects, we created MATLAB MEX functions in C++ that parallelized two OpenSim tasks: (1) calculation of positions and velocities of all contact elements on the bottom of each foot, and (2) calculation of inverse dynamic joint torques. We parallelized OpenSim point kinematic calculations using OpenMP and OpenSim inverse dynamic calculations using MPI. For parallelized point kinematic calculations, inputs were the OpenSim model state; while for parallelized inverse dynamic calculations, inputs were the OpenSim model state and calculated external ground reactions.

Using these parallelized functions, we employed an efficient series of calculation steps each time GPOPS-II needed to evaluate the OpenSim skeletal dynamic equations across all time points. First, we called our parallel C++ point kinematics function to calculate the position and velocity of every contact element in the model. Second, we used this point kinematic information to calculate the contact force acting on each element and the net contact force and torque to be applied to the rear foot and toes segments of each foot. Third, we evaluated our surrogate musculoskeletal geometry using the current joint positions and velocities to find muscle-tendon lengths, velocities, and moment arms for all muscles in the model. Fourth, we used our Hill-type muscle model with the current muscle activations and muscle-tendon lengths and velocities to calculate all tendon forces. Fifth, we calculated muscle contributions to all joint moments by multiplying tendon forces by their associated moment arms. Sixth, we called our parallel C++ inverse dynamics function to apply the calculated ground reactions to the rear foot and toes segments of both feet as OpenSim external forces and calculate inverse dynamic joint torques. Seventh, we calculated joint torque errors for the five muscle-controlled lower-body joints in each leg by subtracting the joint torques calculated in OpenSim using inverse dynamics from the joint torques calculated in MATLAB using GPOPS-II additional controls and moment arms where needed. All of these calculations were performed in MATLAB apart from the two parallel C++ functions that called OpenSim functionality.

#### Calibration Optimizations

We performed two calibration optimizations to determine parameter values in the foot–ground contact models and to provide a starting point for subsequent tracking optimizations (see Table 1, top section). For both optimizations, the cost function tracked experimental marker, ground reaction, and inverse dynamic joint torque data from the periodic trial while also minimizing joint jerk. The constraints enforcing dynamic consistency and joint jerk were the only controls. IPOPT was used in first derivative mode to generate an initial guess, and then SNOPT was started at that initial guess to refine the solution. For the first optimization, static parameters were included for the six coefficients that defined the parabolic distribution of spring stiffness across the bottom of each foot and for the common spring resting length for each foot. For the second optimization, all static parameters were removed from the problem, the parameter values were fixed to those found by the first optimization, and the optimization repeated to verify that identified foot–ground contact model parameter values could closely reproduce the experimentally measured ground reactions for both feet. Root-mean-square (RMS) errors in ground reactions produced by the second optimization were within 10 N for forces and 5 Nm for moments calculated about the midfoot marker projected onto the floor.

#### Tracking Optimizations

Starting from the results of the second calibration optimization, we performed three tracking optimizations for the 0.5 m/s walking speed to provide a starting point for subsequent prediction optimizations (see Table 1, middle section). Each tracking optimization utilized a different method to control 5 lower-body DOFs (hip flexion–extension, hip adduction–abduction, knee flexion–extension, ankle flexion–extension, and ankle inversion–eversion) in each leg. The first tracking optimization was torque-driven (5 independent joint torque controls per leg), the second was activation-driven (35 independent muscle activation controls per leg), and the third was synergy-driven (5 independent synergy controls per leg used to construct the 35 muscle activations per leg). For all three optimizations, the cost function tracked lower-body joint torques (apart from hip internal-external rotation and toes flexion–extension) and upper body, hip internal-external rotation, and toes joint angles from the second calibration optimization while minimizing joint jerk controls. We did not track joint angle and joint torque curves for the same joint to avoid having related terms for the same joint in the cost function. We selected joint angle tracking over joint torque tracking for the toes and upper body joints since we found that large changes in toes or arm motion required only small changes in the corresponding joint torques. We also tracked pelvis angles so that the model would maintain the proper orientation in the laboratory. Path constraints satisfied skeletal dynamics and bounded joint angle errors relative to the second calibration optimization and ground reaction errors relative to experimental data. For the torque-driven optimization, the controls were joint jerk and 5 joint torques for each leg, and algebraic path constraints were used to match 5 inverse dynamic joint torques per leg with the 5 joint torque controls per leg. The activation-driven optimization replaced the 5 torque controls with 35 lower extremity muscle activations per leg, added tracking of 35 activation controls per leg from the EMG-driven models to the cost function, and used algebraic path constraints to match 5 inverse dynamic joint torques per leg with activation controls per leg. The synergy-driven optimization was identical except that the 35 activation controls were replaced with 5 synergy controls per leg, inverse dynamic joint torque matching used synergy-constructed muscle activations, and static parameters were added to allow identification of the corresponding 5 sets of synergy vector weights. In addition, a constraint was added to force each synergy vector to have unit magnitude, making the synergy solutions unique. IPOPT in first derivative mode was used to solve all tracking optimization problems, with gradients calculated using central differencing.

#### Prediction Optimizations

Starting from the results of the tracking optimizations, we performed three prediction optimizations for the 0.5 m/s walking speed as a “sanity check,” three for the 0.8 m/s walking speed as a predictive evaluation, and one using only synergy controls for a 1.1 m/s walking speed as a challenge to our simulation framework. For the 0.5 m/s and 0.8 m/s predictions, the first tracking optimization was torque-driven, the second was activation-driven, and the third was synergy-driven. For the 1.1 m/s prediction, only a synergy-driven model was used to make predictions. The goal of these optimizations was to see whether each type of control could predict not only a realistic walking motion with realistic ground reactions but also the correct period for one gait cycle, which decreases with increasing walking speed. Since the 0.5 m/s speed was the same as the tracking optimization speed, matching the experimental period closely provides a “sanity check” on the solution. Due to problems we encountered with solving these optimal control problems using free final time, we performed each prediction optimization using five values of final time: the average experimental value rounded to the nearest tenth of a second plus or minus 0.1 and 0.2 s. We then fit a parabola to the final cost function values plotted as a function of final time and took the minimum value as the predicted final time. All results reported were taken from the simulations whose final times were closest to the parabola’s minimum value.

For each control situation, the optimal control problem formulation for both speeds was similar to the corresponding tracking optimization except that several cost function and constraint terms were removed and no static parameters were utilized, allowing new walking motions and ground reactions to be predicted. For all three optimizations, the cost function tracked upper body, hip internal–external rotation, and toes flexion–extension joint angles from the corresponding tracking optimization solution while minimizing joint jerk controls, and the path constraints satisfied the skeletal dynamics. The torque-driven problem added tracking of 5 lower extremity joint torques per leg found by the corresponding tracking optimization to the cost function. The activation-driven problem added 35 lower extremity muscle activations per leg to the controls, tracking of 35 activations per leg found by the corresponding tracking optimization to the cost function, and matching of 5 inverse dynamic joint torques per leg with corresponding joint torques produced by muscle activations to the algebraic path constraints. The synergy-driven problem added 5 synergy controls per leg to the controls, tracking of 5 synergy controls per leg found by the corresponding tracking optimization to the cost function, and matching of 5 inverse dynamic joint torques per leg with corresponding joint torques produced by the synergy-constructed muscle activations to the algebraic path constraints. The synergy-driven problem used the synergy vectors found by the corresponding tracking optimization. The implicit assumption in this prediction approach is that when the subject walks at any speed, he will choose controls (joint torques, muscle activations, or synergy controls) that are “close” to those he uses at his self-selected speed of 0.5 m/s. IPOPT in first derivative mode was again used to solve all prediction optimization problems.

## Results

**Figure 2:**
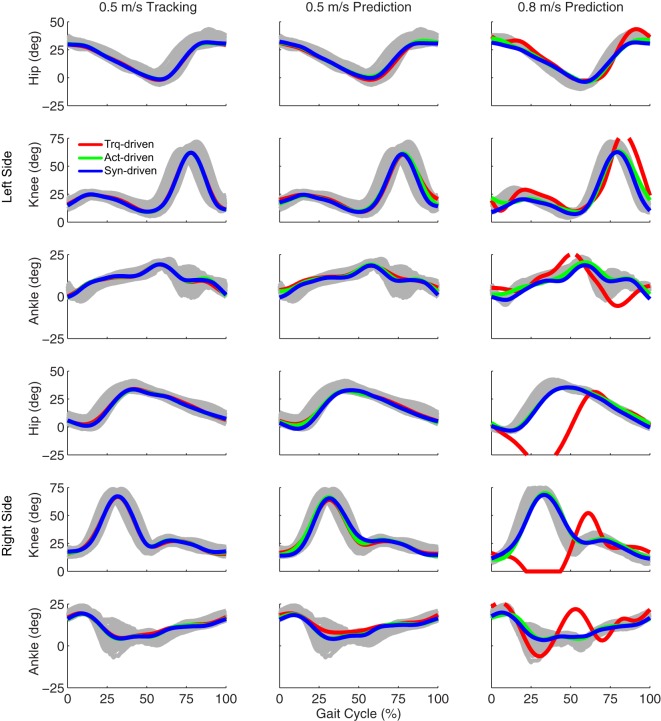
**Sagittal plane lower extremity joint angles from inverse kinematics (gray lines for individual trials), torque-driven optimizations (red lines), activation-driven optimizations (green lines), and synergy-driven optimizations (blue lines)**. First column: results from tracking optimizations at 0.5 m/s. Second column: results from prediction optimizations at 0.5 m/s. Third column: results from prediction optimizations at 0.8 m/s. Top three rows: hip, knee, and ankle angles from left non-paretic leg. Bottom three rows: hip, knee, and ankle angles from right paretic leg.

**Figure 3:**
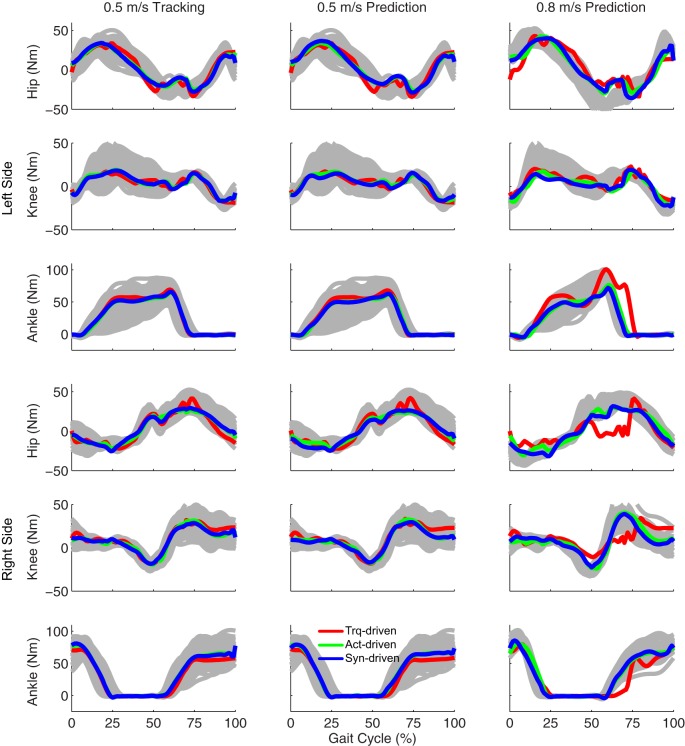
**Sagittal plane lower extremity joint torques from inverse dynamics (gray lines for individual trials), torque-driven optimizations (red lines), activation-driven optimizations (green lines), and synergy-driven optimizations (blue lines)**. First column: results from tracking optimizations at 0.5 m/s. Second column: results from prediction optimizations at 0.5 m/s. Third column: results from prediction optimizations at 0.8 m/s. Top three rows: hip, knee, and ankle torques from left non-paretic leg. Bottom three rows: hip, knee, and ankle torques from right paretic leg.

**Figure 4:**
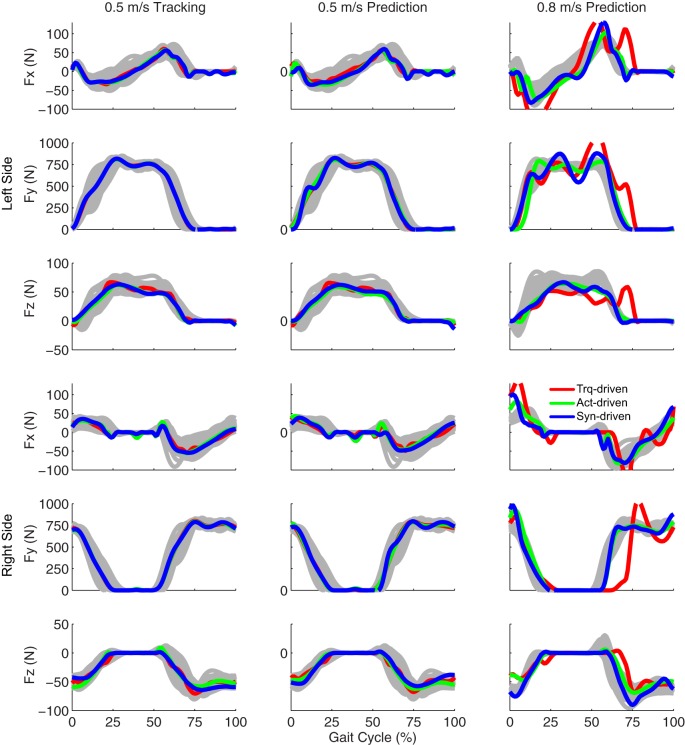
**Ground reaction forces from the experimental data (gray lines for individual trials), torque-driven optimizations (red lines), activation-driven optimizations (green lines), and synergy-driven optimizations (blue lines)**. First column: results from tracking optimizations at 0.5 m/s. Second column: results from prediction optimizations at 0.5 m/s. Third column: results from prediction optimizations at 0.8 m/s. Top three rows: anterior (Fx), superior (Fy), and lateral (Fz) forces from left non-paretic leg. Bottom three rows: anterior (Fx), superior (Fy), and lateral (Fz) forces from right paretic leg.

**Figure 5:**
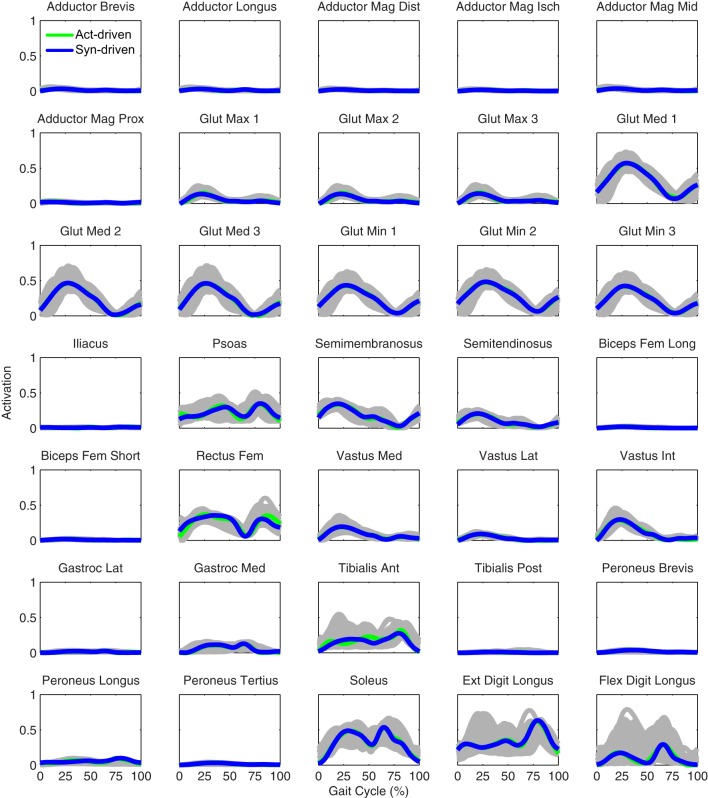
**Left leg muscle activations from the EMG-driven model (gray lines for individual trials), activation-driven tracking optimizations (green lines), and synergy-driven tracking optimizations (blue lines) at 0.5 m/s**. Muscle names are indicated in the figure.

**Figure 6:**
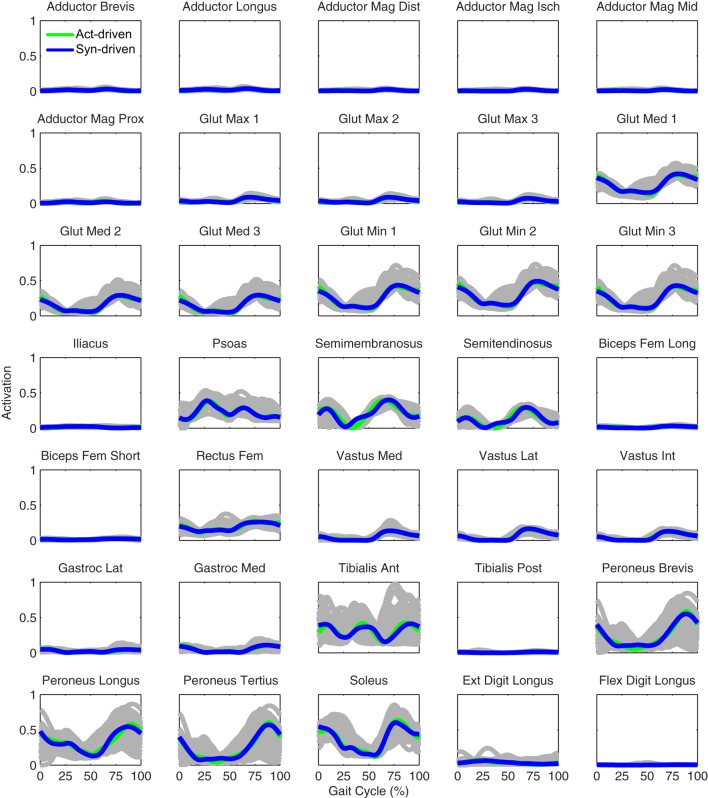
**Right leg muscle activations from the EMG-driven model (gray lines for individual trials), activation-driven tracking optimizations (green lines), and synergy-driven tracking optimizations (blue lines) at 0.5 m/s**. Muscle names are indicated in the figure.

Tracking optimizations using all three types of controls closely reproduced the subject’s experimentally measured walking motion at 0.5 m/s. Simulated lower and upper body joint angles were within experimental ranges from multiple walking cycles (Figure 2 – first column), as were simulated lower-body joint torques (Figure 3 – first column). However, joint torques from the torque-driven model were generally less smooth than those from the activation-driven and synergy-driven models, especially for the two hips. Simulated ground reaction forces were also within experimental ranges (Figure 4 – first column). For the activation-driven and synergy-driven models, simulated activations were within the experimental ranges determined by the EMG-driven models (Figures 5 and 6), with only small changes in activations needed to produce dynamically consistent walking motions.

**Figure 7:**
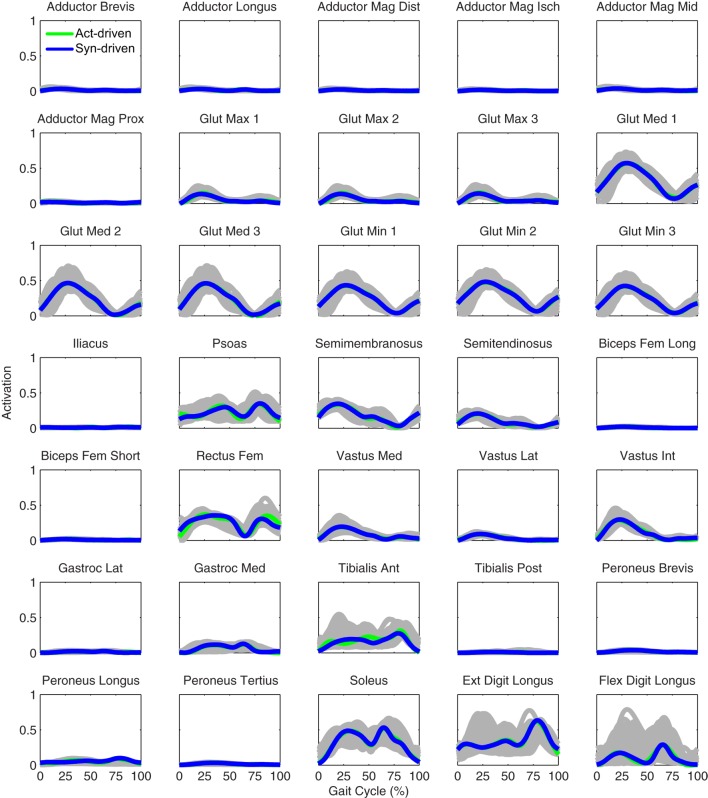
**Left leg muscle activations from the EMG-driven model (gray lines for individual trials), activation-driven prediction optimizations (green lines), and synergy-driven prediction optimizations (blue lines) at 0.5 m/s**. Muscle names are indicated in the figure.

**Figure 8:**
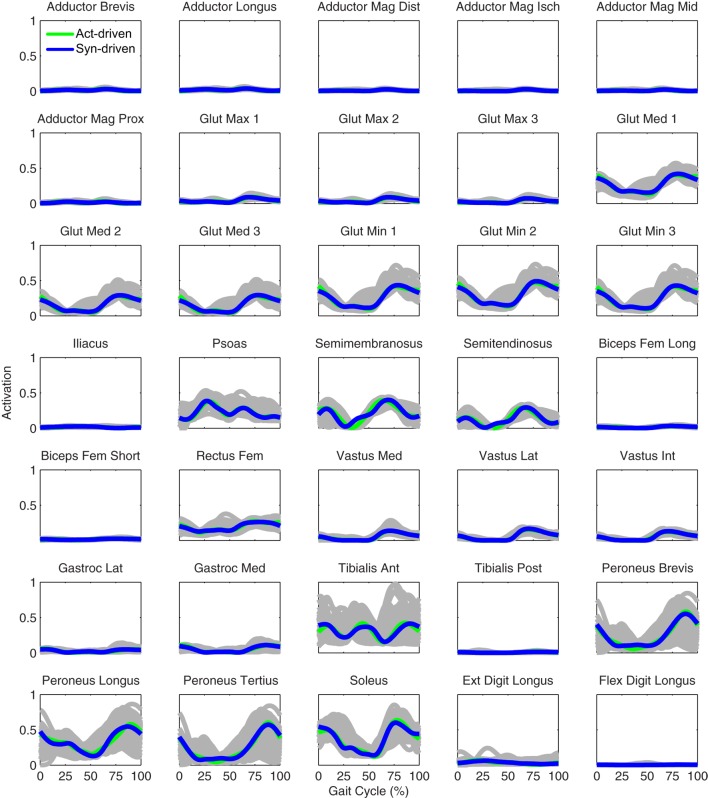
**Right leg muscle activations from the EMG-driven model (gray lines for individual trials), activation-driven prediction optimizations (green lines), and synergy-driven prediction optimizations (blue lines) at 0.5 m/s**. Muscle names are indicated in the figure.

Prediction optimizations using all three types of controls also closely reproduced the subject’s experimentally measured walking motion, gait period, and stride length at 0.5 m/s (Table 2 – top rows). Simulated lower and upper body joint angles (Figure 2 – second column), lower-body joint torques (Figure 3 – second column), and ground reaction forces (Figure 4 – second column) were extremely similar to those produced by the corresponding tracking optimizations. The most noticeable minor differences were for the torque-driven model, where the predicted hip torques were again less smooth than for the other two control types, as were the predicted medial–lateral ground reaction forces. For the activation-driven and synergy-driven models, simulated activations were also very similar to those found by the corresponding tracking optimizations (Figures 7 and 8). All three models predicted a realistic gait period and stride length (Figure 11, first column; Table 2, top rows).

**Table 2:** **Comparison of gait period and stride length between the median experimental walking motions (±experimental ranges) and corresponding predicted walking motions for 0.5 and 0.8 m/s gait speeds**.

	Experiment	Torque prediction	Activation prediction	Synergy prediction
**Gait speed 0.5 m/s**				
Gait period (s)	1.32 ± 0.10	1.35	1.35	1.27
Stride length (m)	0.66 ± 0.05	0.68	0.68	0.64
**Gait speed 0.8 m/s**				
Gait period (s)	1.14 ± 0.06	1.27	1.08	1.16
Stride length (m)	0.91 ± 0.05	1.02	0.86	0.93

*Predicted walking motions were generated using torque-driven, activation-driven, and synergy-driven models. A large difference in gait period and stride length existed between the two experimental gait speeds. All three models predicted gait period and stride length within experimental ranges for the slower speed, while only the synergy-driven model predicted them within experimental ranges for the faster speed*.

**Figure 9:**
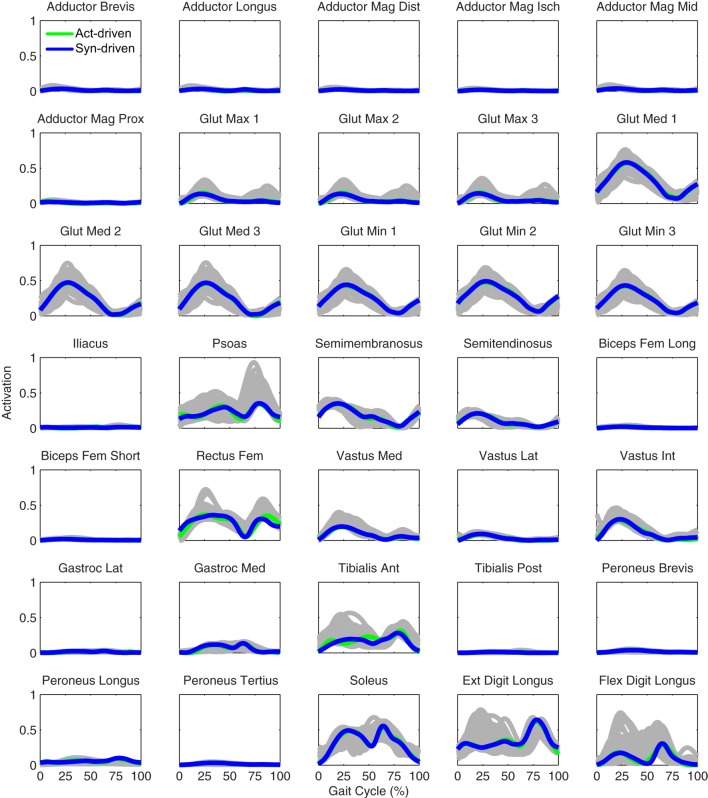
**Left leg muscle activations from the EMG-driven model (gray lines for individual trials), activation-driven prediction optimizations (green lines), and synergy-driven prediction optimizations (blue lines) at 0.8 m/s**. Muscle names are indicated in the figure.

**Figure 10:**
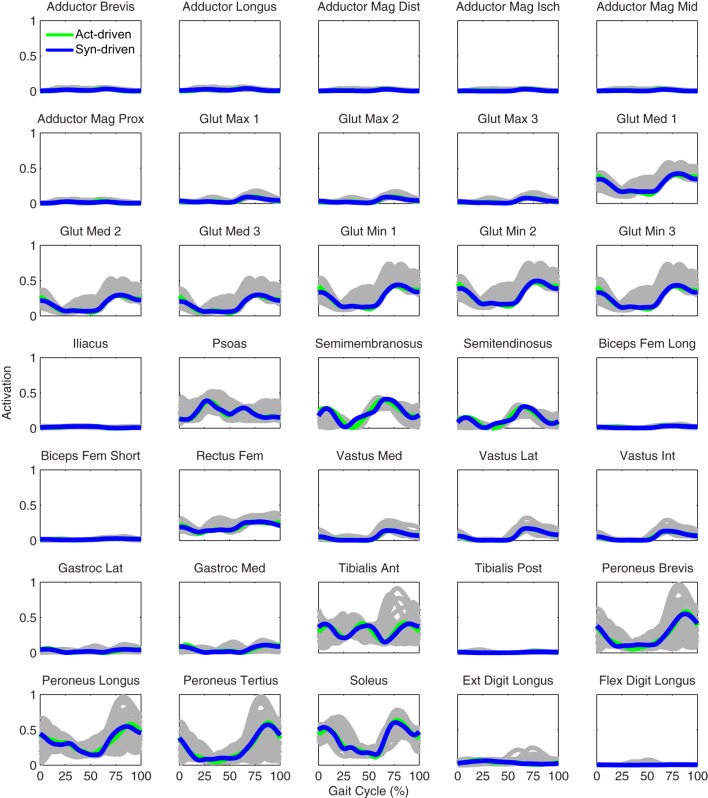
**Right leg muscle activations from the EMG-driven model (gray lines for individual trials), activation-driven prediction optimizations (green lines), and synergy-driven prediction optimizations (blue lines) at 0.8 m/s**. Muscle names are indicated in the figure.

**Figure 11:**
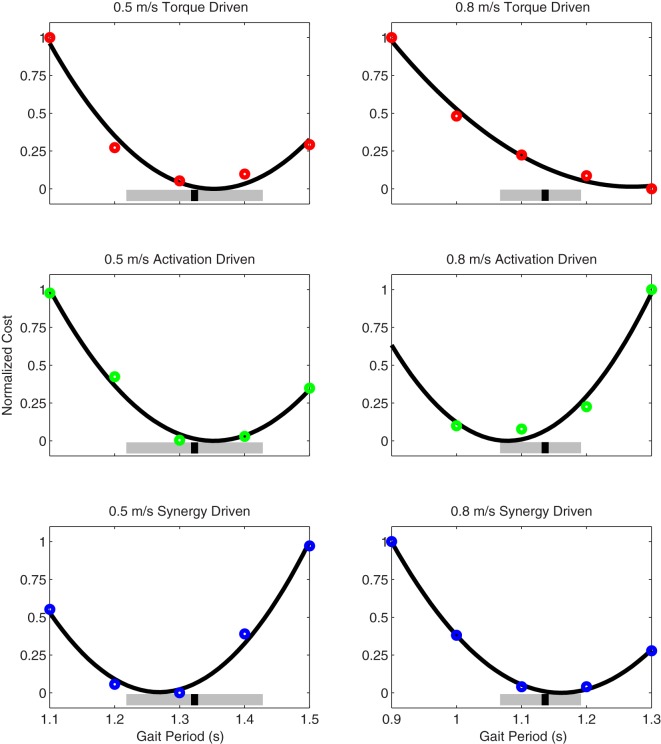
**Normalized cost function values as a function of specified gait period (i.e., final time) as produced by torque-driven (top row – red circles), activation-driven (middle row – green circles), and synergy-driven (bottom row – blue circles) prediction optimizations performed at 0.5 m/s (first column) and 0.8 m/s (second column) walking speeds**. The thick black line in each plot indicates a quadratic fit to the cost function values. The thick gray bars indicate the range of experimentally measured gait periods, and the black hash marks indicate the median gait period. For the activation-driven prediction at 0.8 m/s, the normalized cost function value for a gait period of 0.9 s (not shown) was much higher than expected, likely due to entrapment in a local minimum, and was omitted from the quadratic fit.

**Figure 12:**
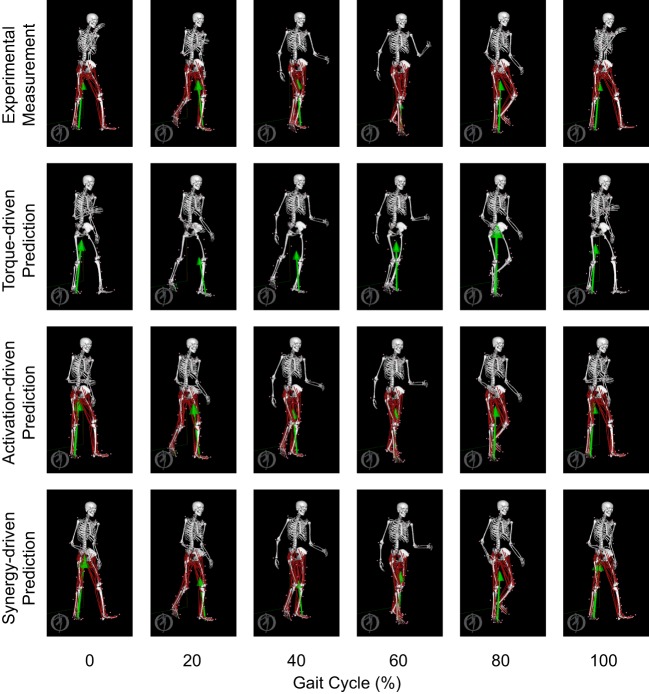
**Animation strips showing walking motions at 0.8 m/s obtained from the most periodic experimentally measured walking trial (first row), the torque-driven prediction optimization (second row), the activation-driven prediction optimization (third row), and the synergy-driven prediction optimization (fourth row)**. Each column represents a different point in the gait cycle starting at 0% on the far left and going to 100% on the far right.

Only prediction optimizations that used activation controls or synergy controls closely reproduced the subject’s experimentally measured walking motion, gait period, and stride length at 0.8 m/s (Table 2 – bottom rows). For these two control types, simulated lower joint angles were within or just outside experimental ranges from multiple walking cycles, while for torque controls, every simulated lower-body joint angle went beyond the experimental ranges (Figure 2 – third column), and the optimization predicted hiking of the paretic hip to compensate for reduced knee flexion on that side, which is a biologically plausible strategy. Interestingly, for all three control types, simulated upper body joint angles were extremely different from those observed experimentally (see Figure 12 for a visual comparison between experimentally measured and computationally predicted full-body motions). Similar to the joint angle results, simulated lower-body joint torques (Figure 3 – third column) and ground reaction forces (Figure 4 – third column) produced by activation and synergy controls generally remained within or at the edge of experimental ranges, while those produced by torque controls generally went beyond them, especially for the ground reaction forces. All three control types predicted an increase in anterior–posterior ground reaction force that was comparable to the increased range measured experimentally to achieve an increase in walking speed. For the activation-driven and synergy-driven models, simulated activations remained within the experimental ranges determined by the EMG-driven models (Figures 9 and 10). The synergy-driven model predicted the most realistic gait period and stride length, which were 1.16 s compared to an experimental median of 1.14 s for gait period and 0.93 m compared to an experimental median of 0.91 m for stride length (Figure 11, second column; Table 2, bottom rows). The gait period and stride length predictions for the activation-driven model were right at the boundary of the measured experimental ranges, while the predictions for the torque-driven model were outside those ranges.

The final prediction optimization used synergy controls to simulate walking at 1.1 m/s, a condition for which no experimental data are available, and predicted a gait period of 1.12 s. Assuming a linear relationship between walking speed and stride length (Jordan et al., 2007), as defined by the subject’s median speed and stride length for 0.5 and 0.8 m/s, the estimated experimental gait period for this faster walking speed would be 1.06 s. Thus, the synergy-driven model predicted not only the best gait period for the 0.8 m/s walking speed but also a physically realistic gait period with physically realistic walking motion (not shown) for an even faster walking speed without available experimental data.

## Discussion

This study developed and evaluated a subject-specific synergy-controlled neuromusculoskeletal simulation framework that predicted three-dimensional walking motions for an individual post-stroke. We investigated whether actuating a neuromusculoskeletal model of the subject with muscle synergy controls (5 per leg) facilitated generation of accurate walking predictions compared to actuating the model with muscle activation controls (35 per leg) or joint torque controls (5 per leg). We found that walking predictions generated for both 0.5 and 0.8 m/s were more accurate (in terms of joint motions, joint torques, ground reactions, and final time) and converged more easily for the activation-driven and synergy-driven models than for the torque-driven model. Furthermore, the accuracy of the walking predictions at both speeds was comparable for the activation-driven and synergy-driven models, even though the synergy-driven model used 30 fewer controls per leg. When the synergy-driven model was used to predict the subject’s walking motion at 1.1 m/s, a condition for which no experimental data were available, the predicted stride length was close to that calculated by the linear speed–stride length relationship fitted to experimental data from 0.5 and 0.8 m/s. Overall, these findings are encouraging and suggest that the current simulation framework could provide a useful foundation for predicting how a patient will interact with different neurorehabilitation approaches (e.g., FES, AFO, exoskeleton, robotic gait trainer, strength training) so that an optimal neurorehabilitation prescription can be identified.

We had to overcome a number of practical challenges to generate the walking predictions presented in this study. One significant challenge was obtaining convergence of our optimal control problems, which we addressed using several strategies. First, we used sequences of optimizations that gradually increased the complexity of the problem being solved and provided a good initial guess for the next level problem. Second, we compared IPOPT in first derivative mode, IPOPT in second derivative mode, and SNOPT to determine which option worked best for our problems. Third, we tracked joint angles rather than joint torques for joints not controlled by muscles, since tracking joint torques permitted large changes in toes and arm motions using only small changes in joint torques. Fourth, we replaced free final time problems with a sequence of five fixed final time problems and then fitted a parabola to the cost function values to determine the final time of the corresponding free final time problem. Fifth, we replaced an explicit dynamics formulation requiring no additional controls with an implicit dynamics formulation requiring additional jerk controls to obtain smooth motion and control predictions. The other significant challenge was obtaining reasonable computation times, which we resolved by parallelizing the computationally costly skeletal dynamics and point kinematics calculations performed by OpenSim. Once a reasonable initial guess was available, torque-driven and synergy-driven problems typically required about 30 min of CPU time to converge, though the variation in convergence time was wide, while activation-driven typically required about an hour of CPU time. On a practical basis, however, we normally perturbed the most recent solution and re-ran each optimization to help avoid entrapment in a local minimum, making estimation of total CPU time difficult. More research is needed to determine how to improve the convergence properties of these problems.

Given that numerous previous studies have generated muscle-actuated full-body forward dynamic simulations of walking (Gerritsen et al., 1998; Anderson and Pandy, 2001; Thelen et al., 2003; Ackermann and van den Bogert, 2010, 2012; Geyer and Herr, 2010; McGowan et al., 2010; Allen et al., 2013; Knarr et al., 2013, 2014; Kia et al., 2014; Dorn et al., 2015), it is worth considering the unique aspects of our approach. Only a small number have predicted new walking motions for which experimental data are not available (Anderson and Pandy, 2001; Ackermann and van den Bogert, 2010, 2012; Dorn et al., 2015). Few studies have used direct collocation optimal control methods to solve for all time points simultaneously (Ackermann and van den Bogert, 2010, 2012), few have used subject-specific (Knarr et al., 2014) rather than scaled generic or simplified musculoskeletal models, and few have modeled individuals with neurological impairment (Allen et al., 2013; Knarr et al., 2013). No previous muscle-actuated full-body walking simulation studies have combined subject-specific EMG-driven modeling with subject-specific synergy controls to define the neural control structure of the model. Furthermore, no previous studies have calibrated joint and ground reaction force parameters in the model to match walking data collected from a specific subject. Thus, the most unique feature of our study was combining all of the various modeling and optimization elements listed above into a single comprehensive simulation framework capable of predicting new walking motions.

Only the activation-driven and synergy-driven models were able to predict the correct walking motion and gait period closely at both 0.5 and 0.8 m/s. Since the only difference between the torque-driven model and the other two models was the control method, it is likely that numerical issues related to the use of pure torque controls were the source of the problem. This hypothesis is supported by results from two previous simulation studies. Risher et al. (1997) showed that even small inconsistencies in inverse dynamic solutions, such as those introduced by spline fitting, can produce large motion errors when the calculated joint torques are used to control a forward dynamic simulation intended to reproduce the original motion. Our approach requires spline fitting of joint torque and joint angle data so that the optimal control solver can obtain values at any desired collocation point. Gerritsen et al. (1998) showed that muscle force–length–velocity properties provide proportional-derivative-like feedback control properties that help stabilize forward dynamic simulations of walking and prevent drift away from a desired motion. Based on the finding of these two studies, it is less surprising that the activation-driven and synergy-driven models performed better than the torque-driven model.

Although both our synergy-driven and activation-driven models generated accurate walking predictions, our synergy-driven model still possesses several distinct advantages. The primary advantage is the significantly reduced number of controls compared to the activation-driven model. By using muscle synergy controls, we were able to predict highly realistic subject-specific walking motions using the same number of controls as in the torque-driven model but without any of the problems encountered by that model. Significantly fewer controls (30 fewer per leg) reduce computational cost and complexity and make the optimal control solution process less sensitive to poor initial guesses. Though not demonstrated in our study, we believe that the synergy-driven model also has the best potential for simulating individuals with neurological impairment, since subject-specific synergy information limits how a subject can coordinate his muscles. For example, it would be interesting to eliminate one synergy at a time from the paretic leg in our model and predict the functional impact on our subject’s walking pattern. Would the model’s ability to reproduce the subject’s walking motion break down if a lower number of synergies were used? Synergy controls within a neuromusculoskeletal model provide an excellent avenue for simulating the functional consequences of reduced neural complexity (Allen et al., 2013).

To use our neuromusculoskeletal simulation framework for actual clinical treatment design, we would need to determine how to incorporate different treatment approaches into the framework. Simulating the effects of strength training could be achieved by increasing the peak isometric strength of individual muscles or groups of muscles. Simulating the effects of FES could be achieved by adding controls to the optimal control problem that augment the activation of one muscle or several muscles. Simulating the effects of an AFO, exoskeleton, robotic gait trainer, or exercise device could be achieved by adding a model of the device to the patient’s OpenSim model [e.g., Fregly et al. (2015)] and adding static parameters and controls to the optimal control problem that account for modifiable design features of the device. The biggest challenge with simulating any of these treatment approaches is defining an appropriate optimization cost function. When predicting how the patient’s neural control system will respond to a treatment, should the cost function minimize an absolute quantity, such as metabolic cost, should it minimize neural control changes away from some experimentally measured baseline situation (as we have done here), or should it hold the patient’s neural control strategy constant and change only static parameters and controls related to the treatment? The issue of predicting how a patient will interact with a treatment is a critical one for researchers to explore in the future.

Despite the high level of subject specificity in our neuromusculoskeletal model, our study possesses a number of limitations that can help inform future research efforts. First, we modeled only a single hemiparetic subject. Our goal for the present study was to develop and evaluate the initial implementation of our neuromusculoskeletal simulation framework, which required use of only a single subject. In the future, we plan to test the framework further using walking data collected from additional hemiparetic subjects. Second, we performed all model calibration steps (lower-body kinematic model, foot–ground contact model, EMG-driven model, muscle synergy model) using only a static trial and walking data, primarily from the subject’s self-selected speed of 0.5 m/s. Though this limitation was planned to simplify the model calibration process, use of a wider variety of calibration movements could improve the predictive capabilities of the model. Third, we did not model any neural feedback mechanisms (e.g., from muscle spindles and Golgi tendon organs). Though the extent to which feedback mechanisms contribute to the control of walking remains controversial, it is possible that inclusion of neural feedback models could have a significant impact on our predicted walking motions (Geyer and Herr, 2010). Fourth, we evaluated our predicted walking motions using only the calibration speed and a single faster non-calibration speed, where the faster speed was only 0.3 m/s faster. A more thorough evaluation would involve a wider range of speeds (our subject was unable to walk comfortably for an extended period of time above 0.8 m/s) and movement tasks. Fifth, while we controlled most lower-body joints with muscles, we tracked experimentally measured joint motions to determine the motion of the toes, hip internal–external rotation, and all upper body joints. Better prediction of toes and upper body motion in particular would likely be achieved if these joints could be controlled by muscles as well. Finally, even though our subject was hemiparetic, we assumed that most model parameter values were the same on both sides of the body, the exceptions being electromechanical delays, EMG scale factors, and muscle synergy control structure. When we allowed model parameter values to be different on the two sides, the accuracy with which our EMG-driven modeling process could predict joint moments improved little, suggesting that our current assumptions about bilateral symmetry were reasonable.

In conclusion, we have presented a novel combination of neuromusculoskeletal and computational modeling approaches that permits prediction of subject-specific walking motions for individuals with neurological impairment. The primary strengths of the approach are the high level of subject-specificity in the neuromusculoskeletal model calibration process, including a subject-specific representation of neural control limitations and capabilities via muscle synergy controls, the flexibility of the framework for changing the optimal control problem formulation and the characteristics of the OpenSim model, the ability to tie muscle synergies to their functional consequences, and the ability to predict new walking motions. With future developments, it may be possible to use our framework to simulate different neurorehabilitation interventions and ultimately to optimize treatment prescription so as to maximize recovery of walking function on an individual patient basis.

## Author Contributions

AM assisted with experimental data collection, processed all experimental data, performed all neuromusculoskeletal modeling work, formulated and ran all optimal control problems, and participated in writing the manuscript draft. IE integrated OpenSim dynamics functionality into the GPOPS-II optimal control environment and helped with revising the manuscript draft. JJ assisted with development and calibration of the foot–ground contact models and helped with revising the manuscript draft. AR assisted with development of optimal control problem formulations, helped troubleshoot GPOPS-II optimal control issues, and helped with revising the manuscript draft. CP recruited the experimental subject, ran the experimental data collection session, provided clinical assessment of predicted walking motions, and helped with revising the manuscript draft. BF planned and supervised the entire project, organized the experimental data collection session, assisted with experimental data collection, directed collaborative efforts between AM, IE, and JJ, assisted with formulation of optimal control problems, evaluated optimal control results, and participated in writing the manuscript draft.

## Conflict of Interest Statement

The authors declare that the research was conducted in the absence of any commercial or financial relationships that could be construed as a potential conflict of interest. The reviewer JS declared a shared affiliation, though no other collaboration, with the authors AM, IE, JJ, AR, CP, and BF to the handling Editor, who ensured that the process nevertheless met the standards of a fair and objective review.

## Supplementary Material

MATLAB code and sample experimental inputs for performing direct collocation optimal control predictions of walking will be made available at https://simtk.org/projects/synwalkingpred.
